# Streptococcus suis Encodes Multiple Allelic Variants of a Phase-Variable Type III DNA Methyltransferase, ModS, That Control Distinct Phasevarions

**DOI:** 10.1128/mSphere.00069-21

**Published:** 2021-05-12

**Authors:** Greg Tram, Freda E.-C. Jen, Zachary N. Phillips, Jamie Timms, Asma-Ul Husna, Michael P. Jennings, Patrick J. Blackall, John M. Atack

**Affiliations:** a Institute for Glycomics, Griffith University, Gold Coast, Queensland, Australia; b Queensland Alliance for Agriculture and Food Innovation, The University of Queensland, St. Lucia, Queensland, Australia; University of Iowa

**Keywords:** R-M systems, *Streptococcus suis*, methyltransferase, phase variation, phasevarion, veterinary pathogen

## Abstract

Streptococcus suis is a significant cause of bacterial meningitis in humans, particularly in Southeast Asia, and is a leading cause of respiratory and invasive disease in pigs. Phase-variable DNA methyltransferases, associated with restriction-modification (R-M) systems, are a source of epigenetic gene regulation, controlling the expression of multiple genes. These systems are known as phasevarions (phase-variable regulons) and have been characterized in many host-adapted bacterial pathogens. We recently described the presence of a Type III DNA methyltransferase in S. suis, ModS, which contains a simple sequence repeat (SSR) tract within the open reading frame of the *modS* gene and which differed in length between individual strains. We also observed that multiple allelic variants of the *modS* gene were present in a population of S. suis isolates. Here, we demonstrate that a biphasic ON-OFF switching of expression occurs in the two most common ModS alleles, ModS1 and ModS2, and that switching is dependent on SSR tract length. Furthermore, we show using single-molecule real-time (SMRT) sequencing that ModS1 and ModS2 are active methyltransferases in S. suis. ON-OFF switching of each ModS allele results in the regulation of distinct phasevarions, with the ModS2 phasevarion impacting growth patterns and antibiotic resistance. This is the first demonstration of a phase-variable Type III DNA methyltransferase in a Gram-positive organism that controls a phasevarion. Characterizing the phenotypic effects of phasevarions in S. suis is key to understanding pathogenesis and the development of future vaccines.

**IMPORTANCE** Streptococcus suis is a causative agent of meningitis, polyarthritis, and polyserositis in swine, and it is a major cause of zoonotic meningitis in humans. Here, we investigate epigenetic gene regulation in S. suis by multiple phasevarions controlled by the phase-variable Type III DNA methyltransferase ModS. This is the first characterized example of a Type III R-M system regulating a phasevarion in a Gram-positive organism. We demonstrate that biphasic ON-OFF switching of ModS expression results in differences in bacterial growth and antibiotic resistance. Understanding the effects of ModS phase variation is required to determine the stably expressed antigenic repertoire of S. suis, which will direct and inform the development of antimicrobial treatments and vaccines against this important pathogen.

## Abstract (precis)

Streptococcus suis is a causative agent of meningitis, polyarthritis, and polyserositis in swine, and it is a major cause of zoonotic meningitis in humans. Here, we investigate epigenetic gene regulation in S. suis by multiple phasevarions controlled by the phase-variable type III DNA methyltransferase ModS.

## INTRODUCTION

Streptococcus suis is an important veterinary pathogen that contributes a substantial disease burden to the swine industry (1). S. suis is also a significant pathogen in humans, causing zoonotic meningitis, most commonly associated with occupational exposure (2) and consumption of contaminated pork products (3). S. suis frequently colonizes the upper respiratory tract of pigs, where it is considered a commensal organism (4). Virulent strains of S. suis are able to adhere to and invade epithelial cells of the airway, allowing access to the bloodstream. In both pigs and humans, S. suis is able to breach the blood-brain barrier, resulting in the development of meningitis and septic shock (4).

Phase variation is the rapid and reversible switching of gene expression, and it plays an important role in the pathogenesis of many organisms, where it is usually associated with expression of surface factors such as capsules (5) and adhesins (6, 7) and with lipooligosaccharide biosynthesis (8). Randomized gene expression as a result of phase variation can complicate vaccine development, as it results in an unstable antigenic repertoire, and is potentially a major cause of vaccine escape. Many bacterial pathogens also encode cytoplasmically located phase-variable DNA methyltransferases, which are associated with restriction-modification (R-M) systems (9). Variable methyltransferase expression results in genome-wide methylation differences, resulting in differential gene regulation by epigenetic mechanisms in systems called phasevarions (phase-variable regulons) (9, 10). Many phasevarions are controlled by the ON-OFF switching of Type III DNA methyltransferases, encoded by *mod* genes. Phasevarions controlled by Type III *mod* genes have been described in Haemophilus influenzae (11, 12), Moraxella catarrhalis (13), Neisseria spp. (14), Helicobacter pylori (15), and Kingella kingae (16). The phasevarions in these human-adapted pathogens all control expression of genes involved in the pathogenesis of these organisms (17–19). All of these phase-variable *mod* genes contain simple sequence repeat (SSR) DNA tracts within their open reading frame. These SSR tracts are highly unstable and are prone to variation in length due to slipped-strand mispairing during DNA replication, resulting in the biphasic ON-OFF switching of gene expression; the *mod* gene is either in-frame and Mod is expressed (ON), or a variation in SSR tract length results in a frameshift and premature stop codon and Mod is not expressed (OFF) (20).

The methyltransferase specificity of a Type III Mod protein is dictated by the central target recognition domain (TRD) of the encoding *mod* gene (9, 10). This TRD differs between the alleles of individual *mod* genes, with the 5′ and 3′ regions of individual *mod* genes being highly conserved between alleles (9, 10). For example, there are 21 *modA* alleles present in nontypeable Haemophilus influenzae (NTHi) and *Neisseria* species, six *modB* and seven *modD* alleles have been identified in Neisseria gonorrhoeae and Neisseria meningitidis, and 19 *modH* alleles are present in H. pylori (10, 21). Our previous analysis of the restriction enzyme database REBASE demonstrated the presence of a *mod* gene in S. suis, which we have named *modS*, that contained a GAGCA_(n)_ SSR tract (22). A follow-up study analyzing a large collection of S. suis isolates determined the presence of three distinct *modS* alleles, each of which methylated an adenine in distinct DNA sequences. ModS1 methylated 5′-GCG^(m6)^**A**DT-3′ (D is either A, G, or T; bold text indicates methylated adenine), ModS2 methylated 5′-VTC^(m6)^**A**TC-3′ (V is either A, G, or C), and ModS3 (present in a single strain identified in GenBank) methylated 5′-GTTC^(m6)^**A**NNNB-3′ (B is either C, G, or T; N is any nucleotide) (23). These specificities were determined through heterologous expression of the methyltransferase in Escherichia coli. While various lengths of GAGCA_(n)_ SSR tracts were present in different strains of S. suis carrying the same *modS* allele, it was not determined whether *modS* is phase variable or whether the ModS protein is an active methyltransferase in S. suis when expressed.

In this study, we investigated phase variation of the two most prevalent *modS* alleles, *modS1* and *modS2*, by enriching populations of S. suis strains for specific GAGCA_(n)_ SSR tract lengths in each encoding *modS* gene. We then determined if differential protein expression occurred in enriched ON-OFF populations of S. suis by using sequential window acquisition of all theoretical mass spectra (SWATH-MS). Clinically relevant phenotypes were assessed *in vitro* to determine if phase variation of the ModS methyltransferase could result in relevant phenotypic changes or advantages *in vivo*. Studying the impact of these systems not only provides an understanding of disease pathogenesis but will also guide the development of vaccines by defining the stably expressed antigenic repertoire of S. suis.

## RESULTS

### ModS is a phase-variable DNA methyltransferase.

**FIG 1:**
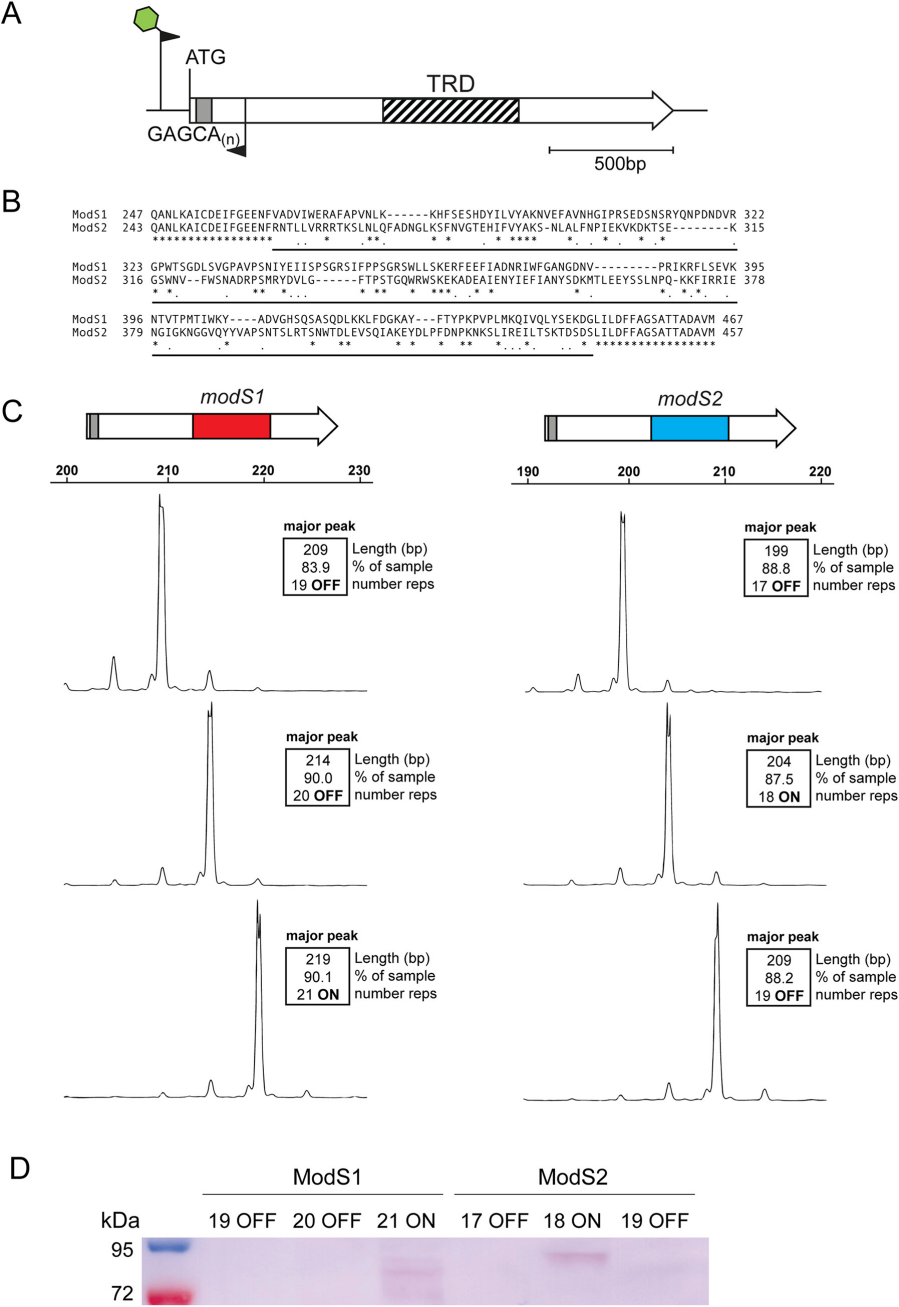
Expression of ModS alleles. (A) The *modS* gene contains a variable-length GAGCA_(n)_ simple sequence repeat (SSR) tract (gray box) near the start of the gene and a variable central target recognition domain (TRD), represented by the hatched box. The 5′ and 3′ regions of the *modS* gene are highly (>95% nucleotide identity) conserved (white). PCR over the SSR tract was determined by 6-carboxyfluorescein (FAM)-labeled PCR using the primers SsuT3-F-FAM and SsuT3-R and analyzed using fragment length analysis. (B) Alignment of the TRD regions of ModS1 and ModS2 showing <25% amino acid identity. An asterisk (*) represents identical amino acid residues; dots (.) represents similar amino acid residues (basic, acidic, and neutral); the TRD region is underlined. Alignments carried out in ClustalW. (C) Fragment length analysis traces of the enriched *modS1* and *modS2* populations of strains LSS89 and SS1056, respectively, containing three consecutive GAGCA_(n)_ SSR tract lengths. (D) Western blot analysis using ModS antiserum demonstrates that the ModS protein is only present in S. suis populations enriched for 21 repeats in ModS1 (LSS89) and 18 repeats in ModS2 (SS1056), demonstrating phase-variable expression of this protein.

In all previous examples of phase-variable Type III *mod* genes, SSR tract length variation results in a biphasic ON-OFF switching of expression. In order to determine if variation in length of the GAGCA_(n)_ repeat tract (Fig. 1A) located in the *modS* open reading frame led to phase-variable expression of the gene, isogenic strains were generated that contained defined lengths of this SSR tract. The isolation of three consecutive repeat tract lengths would theoretically result in one strain where the respective *modS* gene is ON [GAGCA_(n)_ repeat tract length will place the gene in frame and expressed], and two strains of the triplet will be OFF [GAGCA_(n)_ repeat tract length will place the gene out of frame and not expressed]. These enriched populations were generated in S. suis strains LSS89 (*modS1*) or SS1056 (*modS2*), as our analysis of the TRDs of these two alleles showed that they were highly variable and therefore were predicted to methylate different target sequences (Fig. 1B) as we demonstrated previously with heterologous overexpression of ModS1 and ModS2 (23). Strains were enriched for SSR tract lengths containing either 19, 20, or 21 repeats in *modS1* and 17, 18, or 19 repeats in *modS2.* Fragment analysis of each strain confirmed enrichment of each SSR tract length to above 80% (Fig. 1C).

Western blotting of whole-cell lysates of these enriched strains with antiserum generated against the conserved region of all ModS proteins demonstrate that variation in SSR tract length led to a biphasic ON-OFF switching of expression (Fig. 1D; see full blot in Fig. S1 in the supplemental material). The ModS1 protein was only produced in the LSS89 strain, which contained 21 GAGCA repeats in the *modS1* gene. ModS2 was only produced in the SS1056 strain, which was enriched for 18 GAGCA repeats. This matches the prediction from the annotated start codon of both genes (see Fig. S2 in the supplemental material).

10.1128/mSphere.00069-21.1 FIG S1 Expression of ModS is dependent on simple sequence repeat (SSR) tract length. (A) Coomassie staining of whole-cell lysates from enriched strains of S. suis. (B) Western blot using anti-ModS antiserum demonstrates that ModS1 is only produced in strains enriched for 21 GAGCA repeats and that ModS2 is only produced in strains enriched for 18 repeats. The dotted line represents the region of blot presented in Fig. 1D. Download FIG S1, PDF file, 1.7 MB. Copyright © 2021 Tram et al. 2021 Tram et al. https://creativecommons.org/licenses/by/4.0/ This content is distributed under the terms of the Creative Commons Attribution 4.0 International license.

10.1128/mSphere.00069-21.2 FIG S2 Three open reading frames occur in the *modS* gene due to variation in length of the GAGCA_(n)_ simple sequence repeat tract. Due to a frameshift downstream of the GAGCA_(n)_ SSR tract, 19 and 20 repeats result in a premature stop codon (in bold, highlighted with an asterisk [*]), and consequently no expression of the ModS protein; 21 GAGCA_(n)_ repeats in the SSR tract results in the gene being in-frame, and therefore ModS protein is produced. Download FIG S2, PDF file, 0.1 MB. Copyright © 2021 Tram et al. 2021 Tram et al. https://creativecommons.org/licenses/by/4.0/ This content is distributed under the terms of the Creative Commons Attribution 4.0 International license.

To demonstrate methyltransferase activity of the expressed ModS protein, Pacific Biosciences (PacBio) single-molecule real-time (SMRT) sequencing was carried out on genomic DNA isolated from our triplet sets of enriched isogenic strains (19, 20, and 21 repeats for *modS1* in strain LSS89; 17, 18, and 19 repeats for *modS2* in strain SS1056). We detected a separate, distinct Type I methyltransferase motif in strain LSS89 compared to strain SS1056 (Table 1), with these motifs being fully methylated in all three strains of each enriched triplet set. When comparing the methylomes of enriched LSS89 strains, we detected a Type III methyltransferase motif, 5′-GCG**^(m6)^A**T, which was only present in the strain where ModS1 was expressed (21 repeats ON; Table 1). This methylation by ModS1 occurred at 2859 5′-GCG**^(m6)^A**T sites throughout the genome, and was not detected in strains enriched for 19 or 20 GCACA repeats in the *modS1* gene. SS1056 enriched strains exhibited a different Type III motif, 5′-VTC**^(m6)^A**TC, which was only present when ModS2 was expressed (18 repeats ON; Table 1). This motif was methylated 4,810 times in the SS1056 genome, and again was not methylated in strains enriched for 17 or 19 repeats (OFF) in the *modS2* gene. These motifs are the same as those detected previously when these ModS alleles were heterologously overexpressed in E. coli (5).

**TABLE 1:** Summary of methylomes for S. suis strains LSS89 (ModS1) and SS1056 (ModS2)^*a*^

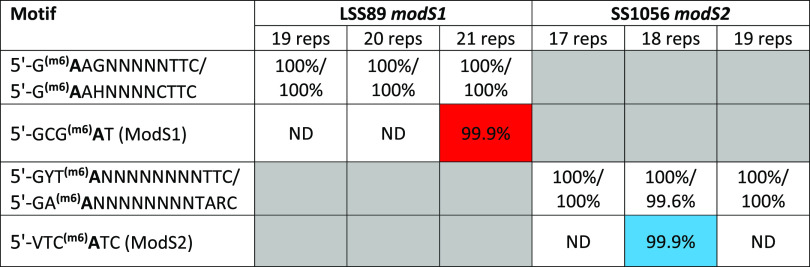

a Strains were enriched for GAGCA_(n)_ SSR tracts of 19, 20, or 21 GAGCA repeats in the *modS1* gene in strain LSS89 or 17, 18, or 19 GAGCA repeats in the *modS2* gene of strain SS1056. Type III methyltransferase motifs are only detected in strains enriched for an ON number of repeats (ModS1 in red in strain LSS89, 21 repeats; ModS2 in blue in strain SS1056, 18 repeats) and match the protein expression detected in Fig. 1. Bold text indicates methylated adenine. In ModS2, V represents A, G, or C. Percentage (%) values represent motifs detected/motifs present. Full methylome data are presented in Data Set S1 in the supplemental material.

10.1128/mSphere.00069-21.3 DATA SET S1 All single-molecule real-time (SMRT) sequencing methylome data summarized in Table 1 for our enriched *modS1* and *modS2* populations of strains LSS89 and SS1056, respectively, containing three consecutive GAGCA_(n)_ SSR tract lengths. Download Data Set S1, XLSX file, 0.01 MB. Copyright © 2021 Tram et al. 2021 Tram et al. https://creativecommons.org/licenses/by/4.0/ This content is distributed under the terms of the Creative Commons Attribution 4.0 International license.

### Biphasic switching of ModS1 and ModS2 results in the expression of distinct phasevarions.

**FIG 2:**
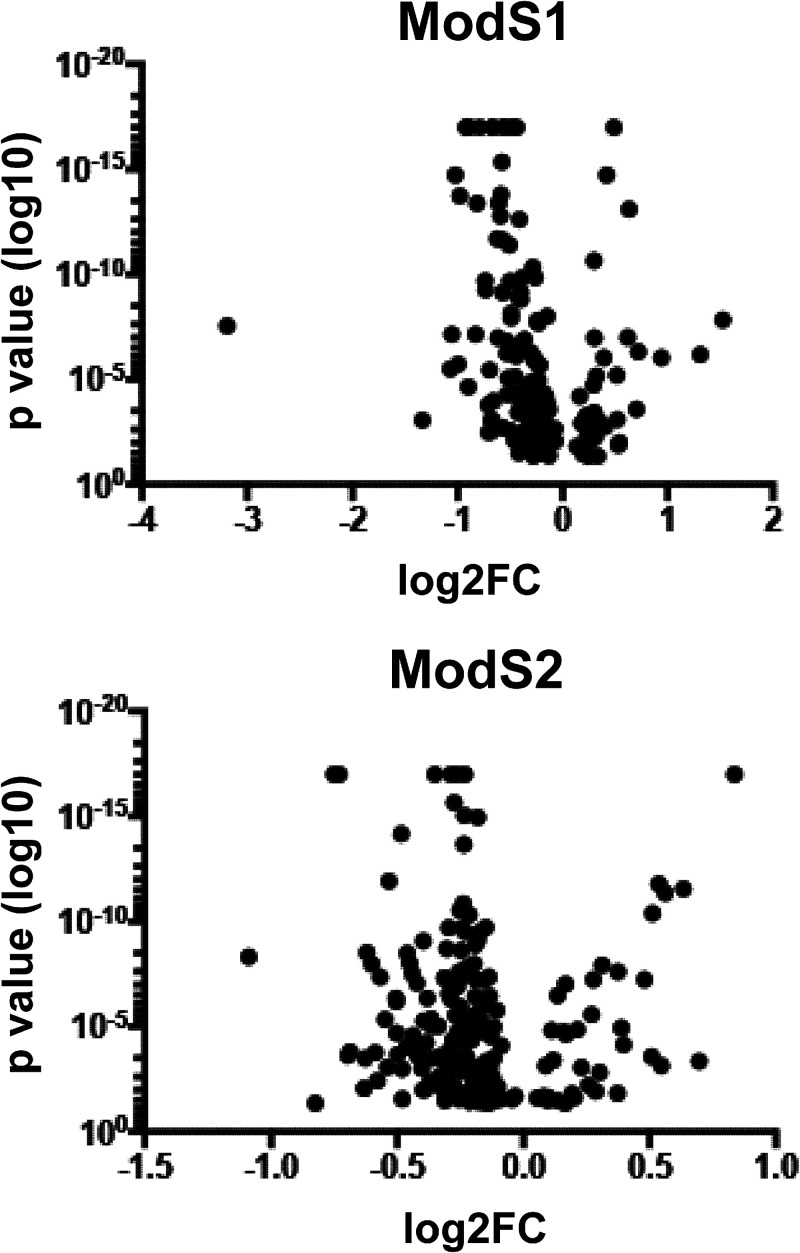
Volcano plot demonstrating changes to protein expression as a result of ModS. SWATH-MS proteomics demonstrated a coverage of 450 of 1,964 identified proteins (∼23%) in the LSS89 ModS1 ON-OFF strain pair, and 411 of 1,905 (∼22%) identified proteins in the SS1056 ModS2 ON-OFF strain pair. The *x* axis indicates relative fold difference in protein abundance in ON compared to OFF; the *y* axis indicates statistical significance.

Quantitative proteomics was carried out to determine if distinct protein expression profiles occurred as a result of ModS expression in S. suis, i.e., if these phase-variable methyltransferases control phasevarions. The expression profiles of an ON-OFF ModS1 pair from strain LSS89 (21 repeats ON and 19 repeats OFF) and an ON-OFF ModS2 pair from strain SS1056 (18 repeats ON and 17 repeats OFF) were assessed using SWATH-MS. Both strain pairs exhibited unique changes in protein expression dependent on phase variation of their respective *modS* gene, with SWATH-MS covering ∼23% of total proteins in LSS89 strains and ∼22% of total proteins in SS1056 strains. Significant differences in protein abundance between the ON and OFF populations are represented as volcano plots in Fig. 2. In strain LSS89, ModS1 ON resulted in higher expression of several proteins involved in a range of cellular processes compared to strains where ModS1 was OFF. Multiple transcriptional regulators were upregulated by expression of ModS1, including the replication initiator protein DnaA (24) and a YlbF/YmcA-type protein (25), as well as transcription factors and repressors. Many ribosomal subunits were upregulated, as well as those involved in general metabolism, such as a nitroreductase (26), glucokinase (27), and a protein involved in alkylphosphonate utilization (28) (Table 2). Conversely, in strains where ModS1 was not expressed, proteins such as ribosomal proteins, several components of ABC transporters, and the fatty acid binding protein DegV (29) were all increased in expression (Table 2).

**TABLE 2:** Differentially regulated proteins (>1.5-fold) in the ModS1 phasevarion

GenPept accession no.	Protein	Fold change^*a*^	*P*
Downregulated in *modS1* ON			
WP_002940030.1	30S ribosomal protein S12	2.87	1.43 × 10^−8^
WP_002938143.1	ABC transporter permease	2.48	6.54 × 10^−7^
WP_009909754.1	50S ribosomal protein L27	1.92	9.04 × 10^−7^
WP_014638653.1	ABC transporter substrate-binding protein	1.65	4.81 × 10^−7^
WP_002936253.1	DegV family protein	1.62	2.53 × 10^−4^
WP_023369127.1	Sugar ABC transporter substrate-binding protein	1.55	7.86 × 10^−14^
WP_004195491.1	50S ribosomal protein L23	1.54	9.68 × 10^−8^
Upregulated in *modS1* ON			
WP_002936659.1	50S ribosomal protein L29	9.14	2.76 × 10^−8^
WP_002940438.1	Amino acid ABC transporter ATP-binding protein	2.1	8.50 × 10^−4^
WP_012027640.1	Ribosome biogenesis GTPase Der	2.08	3.12 × 10^−6^
WP_002942806.1	CsbD family protein	2.03	6.94 × 10^−8^
WP_014636557.1	Transcriptional repressor	1.99	1.78 × 10^−15^
WP_002936247.1	HU family DNA-binding protein	1.97	1.81 × 10^−6^
WP_002936483.1	50S ribosomal protein L7/L12	1.89	1.82 × 10^−14^
WP_002938891.1	Acyl carrier protein	1.88	≤1.00 × 10^−17^
WP_002936048.1	DUF1846 domain-containing protein	1.86	≤1.00 × 10^−17^
WP_014736429.1	Nitroreductase family protein	1.77	2.17 × 10^−5^
WP_014735259.1	30S ribosomal protein S10	1.76	≤1.00 × 10^−17^
WP_002940682.1	30S ribosomal protein S16	1.73	6.91 × 10^−8^
WP_002934959.1	DUF1149 family protein	1.67	4.09 × 10^−14^
WP_023368958.1	Chromosomal replication initiator protein DnaA	1.66	≤1.00 × 10^−17^
WP_023369777.1	dTDP-4-dehydrorhamnose reductase	1.64	1.96 × 10^−10^
WP_002936660.1	50S ribosomal protein L16	1.62	5.40 × 10^−10^
WP_004194840.1	DUF2829 domain-containing protein	1.6	1.60 × 10^−4^
WP_012027178.1	PTS sugar transporter subunit IIA	1.6	3.27 × 10^−3^
WP_002936656.1	50S ribosomal protein L24	1.59	3.52 × 10^−6^
WP_002937025.1	Response regulator transcription factor	1.59	2.89 × 10^−3^
WP_011922043.1	DivIVA domain-containing protein	1.59	8.40 × 10^−4^
WP_002938966.1	YlbF/YmcA family competence regulator	1.58	≤1.00 × 10^−17^
WP_012775074.1	Alkylphosphonate utilization protein	1.54	8.72 × 10^−4^
WP_023370402.1	ROK family glucokinase	1.53	≤1.00 × 10^−17^
WP_002936622.1	30S ribosomal protein S13	1.51	1.00 × 10^−4^
WP_023369315.1	Glycine cleavage system protein H	1.5	1.94 × 10^−12^

a Fold change presented as *modS1* ON versus *modS1* OFF.

In strain SS1056, ModS2 ON-OFF switching resulted in varied expression of a distinct set of proteins (Table 3). ModS2 expression increased the levels of proteins involved in amino acid metabolism such as cysteine synthase and aminopeptidase D (30). Proteins involved in general metabolism, such as dihydroxyacetone kinase (31), also showed increased expression in ModS2 ON. The DNA biosynthesis enzymes ribonucleotide-diphosphate reductase and ribonucleotide-triphosphate reductase (32) were increased in expression in ModS2 ON, as was an acyl carrier protein involved in fatty acid biosynthesis (33). Three proteins were upregulated in strains that did not express ModS2 (OFF), namely, an ATP-dependent Clp protease, the nucleotide exchange factor GrpE, and a glyoxalase/bleomycin resistance/extradiol dioxygenase family protein involved in resistance to antimicrobials.

**TABLE 3:** Differentially regulated proteins (>1.5-fold) in the ModS2 phasevarion

GenPept accession no.	Protein	Fold change^*a*^	*P*
Downregulated in *modS2* ON			
WP_002935876.1	Glyoxalase/bleomycin resistance/extradiol dioxygenase family protein	1.79	≤1.00 × 10^−17^
WP_044673510.1	ATP-dependent Clp protease ATP-binding subunit	1.62	4.35 × 10^−4^
WP_024387015.1	Nucleotide exchange factor GrpE	1.55	2.81 × 10^−12^
Upregulated in *modS2* ON			
WP_012028296.1	50S ribosomal protein L20	2.13	4.82 × 10^−9^
WP_012027287.1	Peptidylprolyl isomerase	1.77	4.40 × 10^−2^
WP_002938891.1	Acyl carrier protein	1.68	≤1.00 × 10^−17^
WP_044754372.1	Cysteine synthase A	1.66	≤1.00 × 10^−17^
WP_002936486.1	50S ribosomal protein L10	1.62	2.35 × 10^−4^
WP_044680965.1	Class 1b ribonucleoside-diphosphate reductase subunit alpha	1.61	1.54 × 10^−4^
WP_044771061.1	Dihydroxyacetone kinase subunit L	1.55	8.68 × 10^−3^
WP_024384331.1	Aminopeptidase P family protein	1.54	3.24 × 10^−4^
WP_044681922.1	Ribonucleoside-triphosphate reductase	1.54	2.97 × 10^−9^
WP_079394259.1	Amino acid ABC transporter substrate-binding protein	1.52	1.07 × 10^−8^
WP_044674536.1	Winged helix-turn-helix transcriptional regulator	1.5	1.70 × 10^−4^

a Fold change presented as *modS2* ON versus *modS2* OFF.

### ModS switching results in differences in growth.

**FIG 3:**
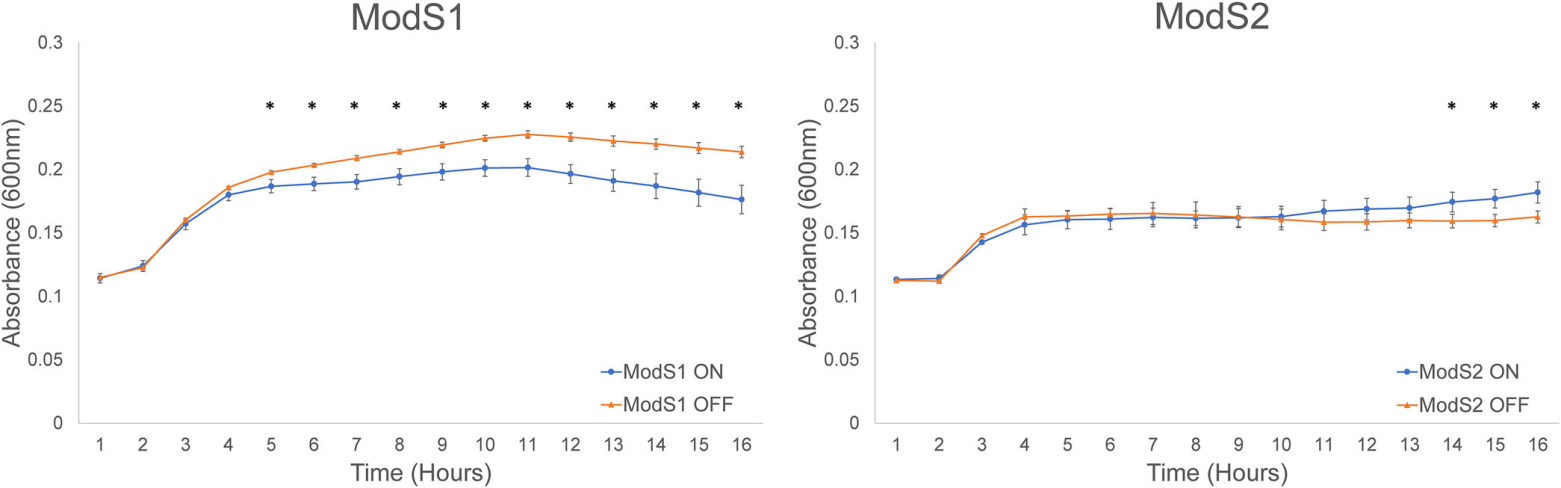
Growth curves of S. suis populations enriched for *modS1* and *modS2*. ON-OFF strain pairs for ModS1 (strain LSS89) and ModS2 (SS1056) were grown in rich medium (THB-Y broth) for 18 h with shaking. Statistically significant differences (*P* < 0.05) in absorbance at each time point are indicated by asterisks, assessed using Student’s *t* test.

Our proteomic analysis of ModS switching demonstrated several proteins that could potentially impact growth rate exhibited differential regulation in both ModS alleles. These included proteins involved in general metabolic enzymes, DNA transcription factors and repressors, and biosynthesis enzymes (Tables 2 and 3). In order to determine if these altered protein expression levels could affect S. suis growth rates, we conducted standard growth curves of our enriched ModS ON-OFF pairs in rich medium. These growth curves showed a small but repeatable (*n* = 3) difference in growth rate for both strain pairs (Fig. 3). Starting at the same optical density at 600 nm (OD_600_), when ModS1 was ON, growth was to a lower final OD (*P* < 0.05), and conversely, when ModS2 was ON, growth was to a higher final OD (*P* < 0.05).

### ModS2 phase variation results in differences in antibiotic resistance.

SWATH-MS also demonstrated differential expression of an antibiotic resistance protein in ModS2 (GenPept accession no. WP_002935876.1), annotated as a glyoxalase/bleomycin resistance/extradiol dioxygenase family protein, which exhibited higher expression in strains which did not express ModS2 (OFF) compared to ModS2 ON. We therefore carried out a MIC analysis using several beta-lactam antibiotics (ampicillin, penicillin, and amoxicillin) and a glycopeptide antibiotic (vancomycin) (Table 4). Although there was no change in MIC to amoxicillin, vancomycin, and penicillin, there was a 2-fold increase in resistance to ampicillin when ModS2 was OFF compared to ModS2 ON (0.32 μg/ml versus 0.16 μg/ml).

**TABLE 4:** MICs of ModS2 ON versus ModS2 OFF enriched S. suis strains

Drug	MIC (μg/ml) for S. suis:	
	ModS2 ON	ModS2 OFF
Ampicillin	0.16	0.31
Vancomycin	1.25	1.25
Amoxicillin	0.16	0.16
Penicillin	0.63	0.63

## DISCUSSION

Over the last 15 years, phasevarions (phase-variable regulons) controlled by ON-OFF switching of Type III *mod* genes have emerged as an important gene regulation strategy in a diverse range of host-adapted pathogens (9, 12, 13, 15, 16, 21). In all of these examples, phase-variable ON-OFF switching of *mod* expression occurs via changes in the length of locus-encoded SSR tracts. In this study, using a combination of strain enrichment, Western blotting, and SMRT sequencing, we conclusively demonstrate that S. suis contains multiple allelic variants of a new phase-variable Type III *mod* gene, *modS*, with this representing the first example of a phasevarion controlled by biphasic ON-OFF switching of a Type III DNA methyltransferase in a Gram-positive organism (Fig. 1). Utilizing an antiserum that recognizes all ModS alleles with equal affinity (9, 34), it was found that both ModS alleles were only expressed in strains with specific SSR tract lengths. ModS1 was only present in strains with 21 GAGCA repeats, and ModS2 was only expressed in strains with 18 GAGCA repeats. In both cases, the repeat length placed the *modS* gene in the correct reading frame and demonstrates that the expression of the ModS protein is dependent on the SSR tract length.

SMRT sequencing was used to determine methyltransferase activity and specificity of ModS1 and ModS2. LSS89 strains of S. suis enriched for ModS1 expression (21 GACAC repeats ON) exhibited methylation of the adenine in 5′-GCG**^m6^A**T motifs, which was not seen in strains in which ModS1 was OFF (19 or 20 GACAC repeats). ModS2-expressing strains (SS1056) of S. suis exhibited methylation of a Type III Mod motif of 5′-VTC**^m6^A**TC motifs (where V can be either A, G, or C) when ModS2 was predicted to be ON (18 GACAC repeats). This confirmed our previous findings when we overexpressed ModS1 and ModS2 using a recombinant E. coli system (23). SMRT sequencing also demonstrates that both ModS1 and ModS2 are active methyltransferases in S. suis, methylating at thousands of sites in their respective genomes (in strain LSS89, 2,861 5′-GCG**^m6^A**T sites are present; in SS1056, 4,812 5′-TC**^m6^A**TC sites are present; see Data Set S1 in the supplemental material).

SWATH-MS proteomic analysis confirmed that the altered methylation resulting from ModS phase variation results in a change in the expression of numerous proteins in distinct phasevarions. These changes in protein expression were seen across many families of protein, including those involved in central metabolism, gene regulation, and transporters. Although phase variation can be a frequent cause of vaccine escape across bacterial species (20), our investigation shows that no proteins currently investigated as vaccine targets are subject to variation by ModS1 or ModS2 in the strains tested (35, 36). By randomly switching expression of different sets of genes, phasevarions allow generation of multiple phenotypic states within a bacterial population. This gives the population an extra contingency strategy for survival, as some phenotypes may be more beneficial under certain environmental conditions, or give certain individual bacteria a particular advantage, allowing survival of the population through selection bottlenecks. Phase-variable switching of ModS resulted in differences in growth rate, but it is unclear whether this is a result of an alteration of regulatory genes due to ModS switching, or another, as-yet-uncharacterized effect of ModS phase variation. When strain pairs were started at the same seeding density, although the final differences in final OD between enriched ON and OFF strain pairs were small, they are statistically significant (*P* < 0.05) and could have an effect on both long-term carriage and disease, particularly if one population has an advantage resulting from differential protein expression over time. ModS2 phase variation also resulted in a difference in antibiotic resistance. A protein repressed by ModS2 expression, annotated as a glyoxalase, bleomycin resistance, and extradiol dioxygenase family protein, has been described as involved in resistance to beta-lactam and glycopeptide antibiotics (37), which are commonly used to treat bacterial infections in humans as well as commercial pig farms (38). The MIC of S. suis was assessed against three commonly used beta-lactams (ampicillin, amoxicillin, and penicillin) and a glycopeptide from the same antibiotic class as bleomycin (vancomycin). A strain in which ModS2 was OFF, where the expression of this resistance protein was increased, showed a small (2-fold) increase in MIC toward ampicillin compared to that of the isogenic strain in which ModS2 was ON. Although small, there may be a cumulative effect, particularly as many beta-lactam antibiotics are extensively used prophylactically in commercial pig farms (39–42). This has the potential to result in a long-term increase in ampicillin resistance in S. suis populations, although this would need to be studied in a model system and is beyond the scope of this work.

Although many of the changes in protein expression determined by our SWATH-MS analysis of enriched ON-OFF population in each ModS allele were small (1.5-fold to 2-fold), SWATH-MS is a technique which quantitates protein expression based on abundance (43). Differential expression of proteins will therefore only identify differences in highly expressed proteins, and expression differences of proteins beyond the limit of detection will be missed. We recognize that this is a limitation of our approach, but a SWATH-MS proteomics approach has been previously used to characterize phasevarion-mediated changes to protein expression (13, 44), with similar small differences in expression also reported in these phasevarions. Therefore, while we have determined that both ModS1 and ModS2 affect the expression of small distinct sets of proteins, it is likely that further changes to gene and protein expression remain undetected. In order to thoroughly characterize each phasevarion, further analysis will be needed. It will also be important to characterize the phasevarions controlled by other *modS* alleles; in previous work we showed that a third *modS* allele—*modS3*—was present in S. suis. The ModS3 allele methylated a distinct sequence to ModS1 and ModS2 (23) and is therefore highly likely to control a different phasevarion. The presence of additional *modS* alleles should also be determined and studied, in order to detail all gene and protein expression changes mediated by phasevarions in S. suis.

This characterization of ModS is the first described instance of a phasevarion in a Gram-positive organism controlled by a Type III methyltransferase. Different alleles of ModS methylate distinct motifs and result in distinct phasevarions. These distinct ModS phasevarions result in gross differences in growth rate and, in the case of ModS2, differential resistance to antibiotics. Both of these phenotypes could affect disease and pathology, and this remains to be studied using both *in vitro* models and, ideally, an *in vivo* challenge using the natural host (pigs). A thorough understanding of phase variation of gene expression, and in particular of phasevarions, is required in order to determine the stably expressed antigenic repertoire of a bacterial species. The prevalence of phase-variable methyltransferases across the bacterial domain demonstrates that phasevarions are a widespread contingency strategy (22, 45, 46) and that characterization of these systems is imperative in order to rationally design effective vaccines that only target stably expressed antigens in the organisms where they are present.

## MATERIALS AND METHODS

### Bacterial strains and growth.

S. suis strains LSS89 and SS1056 (47) used in this study were grown in Todd-Hewitt broth (THB; Oxoid) supplemented with 2% yeast extract (THB-Y) or on THB-Y plates (THB-Y supplemented with 1.5% wt/vol agar). Cultures were incubated overnight at 37°C.

### Strain enrichment.

Fragment length analysis of the SSR tract in each *modS* allele was conducted to determine the length of the GAGCA_(n)_ SSR tract. A PCR was performed using a fluorescently labeled forward primer SsuT3-F-FAM (5′-FAM-CAT CAA AAA CGG CTT GAC AGC C) and the reverse primer SsuT3-R (5′-GCA ATG TTG TCT GAT AAA ACA TCT TTT G), as described previously (23). DNA fragment length analysis was carried out at the Australian Genome Research Facility (AGRF; Brisbane, Australia). This technique was used to enrich populations of S. suis strains for defined SSR lengths through a combination of fragment length analysis and subculturing. S. suis populations of strain LSS89 (encoding *modS1*) were enriched for 19, 20, and 21 GAGCA repeats, and strain SS1056 (encoding *modS2*) were enriched for 17, 18, and 19 GAGCA repeats. Populations containing >80% of each single tract length were considered to be enriched and were used in subsequent studies.

### SDS-PAGE and Western blot analysis.

Cell lysates of each enriched strain were prepared from two 25-ml overnight liquid cultures of S. suis cells grown in THB-Y broth and pooled. The absorbance of each culture was assessed, and samples were then normalized by centrifugation and resuspension to a calculated OD_600_ of 30.0 in 1 ml of radioimmunoprecipitation assay (RIPA) buffer (150 mM NaCl, 1% Triton X-100, 0.5% SDS, and 50 mM Tris HCl [pH 8]). Samples were sonicated twice for 30 s at a probe intensity (amplitude) of 15 μm, on ice. Lysates were clarified by centrifugation at 14,000 × *g* for 20 min to remove cellular debris.

Supernatants were prepared for SDS-PAGE using BOLT 4× loading dye containing 100 mM dithiothreitol (Sigma) and boiled for 30 min, then loaded on precast 4 to 12% BOLT Bis-Tris gel (Thermo Fisher) and run for 45 min at 165 V in morpholinepropanesulfonic acid (MOPS) 1× running buffer according to the manufacturer’s instructions (Thermo Fisher). Proteins were visualized by Coomassie brilliant blue staining. Twice the amount of lysate was loaded onto gels for use in Western blotting, and proteins were transferred to preequilibrated polyvinylidene difluoride (PVDF) membranes (Bio-Rad) at 20 V for 60 min using the BOLT transfer system (Thermo Fisher). Following transfer, membranes were blocked with 10% skim milk in Tris-buffered saline with Tween 20 (TBST) for 60 min. Membranes were incubated with a 1:1,000 dilution of anti-ModS antiserum (raised as detailed below) overnight at 4°C, followed by a 1:10,000 anti-mouse alkaline phosphatase secondary antibody (Sigma). Blots were developed using nitro-blue tetrazolium (NBT)/5-bromo-4-chloro-3′-indolyphosphate (BCIP) (Roche) according to the manufacturer’s instructions.

### Construction of a TRD-less ModS protein.

The *modS1* gene was amplified from S. suis strain LSS89, excluding the CAGCA_(n)_ SSR tract, using the primers SsuT3-oE-F (5′-AGTCAG CATATG AGC AGA GCA AAG CAA AAG CTT GGA GAA TAC ACT CAA G; underlined text represents NdeI site for cloning) and SsuT3-oE-R (5′-AGTCAG GGATCC CTA CAC CAC CTT CAC TTT GGT ACC; underlined text represents BamHI site for cloning) with KOD Hot Start DNA polymerase (Merck Millipore) according to the manufacturer’s instructions. The resulting product was then cloned into the NdeI-BamHI site of the expression vector pET15b (Novagen) with the gene in-frame with the N-terminal His tag to generate vector pET15b:SsuT3-His_tag. The TRD coding region of *modS* was then removed, and the conserved 5′ and 3′ coding regions were fused. This was generated by using the pET15b::SsuT3-His_tag vector as a template via inverse PCR with the primers SsuT3-TRD-remove-F (5′-CCCA AAT ATC TCA TCA CAG ATA GCT TTG AGG TTG G) and SsuT3-TRD-remove-R (5′-GCT GTT ATG CAG CTG AAT GCA GAA GAT GG) binding either side of the TRD, using KOD Hot Start DNA polymerase (Merck Millipore) according to the manufacturer’s instructions. His-tagged TRD-less ModS was overexpressed in E. coli BL21 using isopropyl β-d-1-thiogalactopyranoside (IPTG) induction (0.5 mM) overnight at 37°C with 200 rpm shaking, and protein purified using Talon resin (TaKaRa Bio) using standard protocols in 50 mM phosphate buffer (pH 7.4) containing 300 mM NaCl.

### Generation of ModS antiserum.

A cohort of five 6- to 8-week-old female BALB/c mice (Animal Resources Centre, WA, Australia) were immunized subcutaneously with 25 μg of recombinant TRD-less ModS protein in 25 μl phosphate-buffered saline (PBS), mixed with 25 μl Freund’s adjuvant (Freund’s complete adjuvant [FCA] on day 0 and Freund’s incomplete adjuvant [FIA] subsequently on days 14, 21, 28, and 42; Merck, Darmstadt, Germany). Terminal bleeds were collected on day 58, and serum was separated via centrifugation. Preimmune (naive) serum was collected from cohorts prior to immunization via tail bleed. Serum was stored in 50% glycerol at −20°C. This antiserum recognizes all ModS alleles with equal affinity, as it was raised against the conserved regions of ModS shared by all alleles. All animal work was carried out according to the Australian Code for the Care and Use of Animals for Scientific Purposes, with approval from the Griffith University Animal Ethics Committee (GLY/16/19/AEC).

### Single-molecule real-time (SMRT) sequencing and methylome analysis.

Genomic DNA from our enriched triplet sets of S. suis strains LSS89 (*modS1* 19, 20, and 21 repeats) and SS1056 (*modS2* 17, 18, and 19 repeats) was prepared from an overnight culture in THB-Y broth, and high-molecular-weight genomic DNA was isolated using the Sigma GenElute kit (Sigma-Aldrich) according to the manufacturer's instructions. SMRT sequencing and methylome analysis was carried out as previously described (48, 49). Briefly, DNA was sheared to an average length of approximately 5 to 10 kb (genomic DNA) using g-Tubes (Covaris, Woburn, MA), and SMRTbell template sequencing libraries were prepared using sheared DNA. DNA was end repaired, then ligated to hairpin adapters. Incompletely formed SMRTbell templates were degraded with a combination of exonuclease III (New England Biolabs; Ipswich, MA) and exonuclease VII (USB; Cleveland, OH). Primer was annealed and samples were sequenced on the PacBio Sequel system (Menlo Park, CA) using standard protocols for long insert libraries. SMRT sequencing and methylome analysis was carried out at SNPSaurus (University of Oregon, OR, USA).

### SWATH-MS proteomics.

Overnight cultures of each S. suis strain (10^7^ CFU/ml) were harvested, lysed in guanidium buffer (6 M guanidium chloride, 50 mM Tris-HCl [pH 8], and 10 mM dithiothreitol) and incubated at 30°C for 30 min with shaking (500 rpm). Cysteines of the total protein were alkylated by addition of acrylamide to a final concentration of 25 mM and incubated at 30°C for 60 min with shaking (500 rpm). Concentration of samples was assessed using a NanoDrop 2000 spectrophotometer (Thermo Fisher). A 100-μg aliquot of the protein was then precipitated by addition of 1:1 methanol:acetone at −20°C overnight. The protein was pelleted at 18,000 × *g* for 10 min, and supernatant was removed before the pellet was resuspended in 50 μl trypsin reaction buffer,1 μg trypsin (New England Biolabs) was added, and the suspension incubated overnight at 37°C. Tryptic-digested peptides were then desalted and purified using a ZipTip (Millipore) per the manufacturer’s instructions. SWATH-MS was performed as previously described (50). Briefly, tryptic peptides were analyzed by liquid chromatography-electrospray ionization-tandem mass spectrometry (LC-ESI-MS/MS) using a Prominence nanoLC system (Shimadzu) and a TripleTOF 5600 mass spectrometer with a NanoSpray III interface (Sciex). Peptides were separated on a Vydac Everest reversed-phase C_18_ high-performance liquid chromatography (HPLC) column at a flow rate of 1 μl/min. A gradient of 10% to 60% buffer B over 45 min, with buffer A (1% acetonitrile and 0.1% formic acid) and buffer B (80% acetonitrile and 0.1% formic acid) was used. A mass spectrometry (MS)-time of flight (TOF) scan was performed from an *m/z* range of 350 to 1,800 for 0.5 s, followed by information-dependent acquisition of MS/MS of the top 20 peptides from *m/z* 40 to 1,800 for 0.05 s per spectrum, with automated CE (capillary electrophoresis) selection. Identical LC conditions were used for SWATH-MS. SWATH-MS of triplicate biological replicates was performed with the same MS-TOF scan, followed by high-sensitivity information-independent acquisition with *m/z* isolation windows with 1 *m/z* window overlap each for 0.1 s across an *m/z* range of 400 to 1,250. Collision energy was automatically assigned by Analyst software (AB Sciex) based on *m/z* window ranges. Proteins were identified by searching against S. suis Lss89 and SS1056 genomes (NCBI accession no. GCA_900059105.1 and GCA_900051945.1, respectively) and common contaminants with standard settings using ProteinPilot 5.0.1 (AB Sciex). False-discovery-rate analysis was performed on all searches. ProteinPilot search results were used as ion libraries for SWATH analyses. The abundance of proteins was measured automatically using PeakView (AB Sciex) with standard settings. Comparison of protein relative abundance was performed based on protein intensities or ion intensities using a linear mixed-effects model with the *MSstats* package in R. Proteins with ≥1.5-fold changes in abundance and with adjusted *P* values greater than ≤0.05 were considered differentially expressed.

### Growth curves and MIC assays.

Growth curves were conducted by dilution of overnight cultures to an OD_600_ of 0.1. These cultures were then used to seed a 96-well plate in triplicate. Plates were then incubated at 37°C with shaking in a Tecan Infinite 200 Pro plate reader, with absorbance readings (OD_600_) being taken every 15 min.

The MIC was measured by broth microdilution in triplicate experiments based on CLSI guidelines as described previously (51). Briefly, an overnight culture of S. suis was diluted to an OD_600_ of 0.1 and subcultured grown to the mid-log phase for 3 h at 37°C. Mid-log-phase cultures were diluted to an OD_600_ of 0.2, and 50 μl of each culture was added to 96-well plates containing serially diluted antibiotic concentrations (5 to 0.08 μg/ml), and plates were grown at 37°C with 5% CO_2_ for 24 h. The MIC (mg/liter) was determined as the last dilution at which turbidity was observed following overnight growth, with all assays being performed in triplicate.

### Data availability.

The mass spectrometry proteomics data have been deposited to the ProteomeXchange Consortium via the PRIDE (52) partner repository under data set identifier PXD023726.

10.1128/mSphere.00069-21.4 DATA SET S2 All data for growth curves presented in Fig. 3. Download Data Set S2, XLSX file, 0.01 MB. Copyright © 2021 Tram et al. 2021 Tram et al. https://creativecommons.org/licenses/by/4.0/ This content is distributed under the terms of the Creative Commons Attribution 4.0 International license.
