# Supplementary figures and images for: Streptococcus suis Encodes Multiple Allelic Variants of a Phase-Variable Type III DNA Methyltransferase, ModS, That Control Distinct Phasevarions
Source: mSphere. 2021 May 12;6(3):e00069-21. doi: 10.1128/mSphere.00069-21 (PMC8125046; doi:10.1128/mSphere.00069-21)

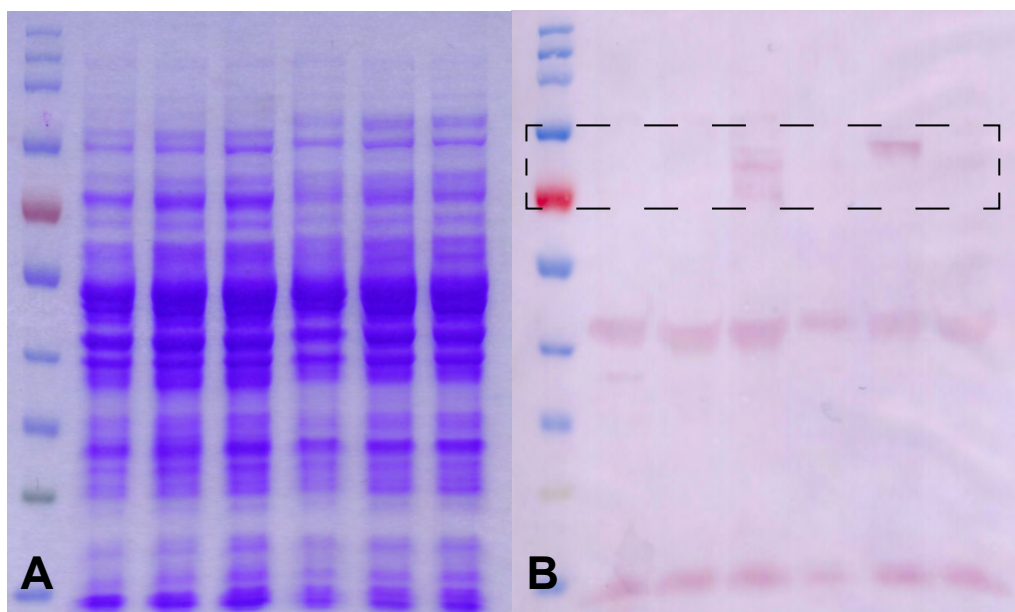

**Supplementary Figure 1.**

Supplement: FIG S1 [file mSphere.00069-21-sf001.pdf]
