# Supplementary material for: Streptococcus suis Encodes Multiple Allelic Variants of a Phase-Variable Type III DNA Methyltransferase, ModS, That Control Distinct Phasevarions
Source: mSphere. 2021 May 12;6(3):e00069-21. doi: 10.1128/mSphere.00069-21 (PMC8125046; doi:10.1128/mSphere.00069-21)
Supplement: FIG S2 [file mSphere.00069-21-sf002.pdf]

19 GAGCA (OFF)

AT**GAGCAGAGCAGAGCAGAGCAGAGCAGAGCAGAGCAGAGCAGAGCAGAGCAGAGCAGAGCAGAGCAGAGCAGAGCAGAGCAGAGCAGAGCAGAGCA**GAAGCTTGGAGACTACACTCAAGTTGAAAAGC***TAG***ACCTAAGGTCAAAAGATGTTTTA >
M S R A E Q S R A E Q S R A E Q S R A E Q S R A E Q S R A E Q S R S L E T T L K L K S *

20 GAGCA (OFF)

AT**GAGCAGAGCAGAGCAGAGCAGAGCAGAGCAGAGCAGAGCAGAGCAGAGCAGAGCAGAGCAGAGCAGAGCAGAGCAGAGCAGAGCAGAGCAGAGCAGAGCA**GAAGCTTGGAGACTACACTCAAGT***TGA***AAAGCTAGACCTAAGGTCAAAAGATGTTTTA >
M S R A E Q S R A E Q S R A E Q S R A E Q S R A E Q S R A E Q S R A E A W R L H S S *

21GAGCA (ON)

AT**GAGCAGAGCAGAGCAGAGCAGAGCAGAGCAGAGCAGAGCAGAGCAGAGCAGAGCAGAGCAGAGCAGAGCAGAGCAGAGCAGAGCAGAGCAGAGCAGAGCAGAGCA**GAAGCTTGGAGACTACACTCAAGTTGAAAAGCTAGACCTAAGGTCAAAAGATGTTTTA >
M S R A E Q S R A E Q S R A E Q S R A E Q S R A E Q S R A E Q S R A E Q K L G D Y T Q V E K L D L R S K D V L >

Supplementary Figure 2.
